# Dihydroxyacid dehydratase is important for gametophyte development and disruption causes increased susceptibility to salinity stress in *Arabidopsis*

**DOI:** 10.1093/jxb/eru449

**Published:** 2014-11-13

**Authors:** Chun Zhang, Qiuying Pang, Luguang Jiang, Shoucai Wang, Xiufeng Yan, Sixue Chen, Yan He

**Affiliations:** ^1^National Maize Improvement Centre of China, Beijing Key Laboratory of Crop Genetic Improvement, China Agricultural University, Beijing, China; ^2^Alkali Soil Natural Environmental Science Centre, Key Laboratory of Saline-alkali Vegetation Ecology Restoration in Oil Field, Northeast Forestry University, Harbin, Heilongjiang, China; ^3^Department of Biology, Genetics Institute, and Plant Molecular & Cellular Biology Program, University of Florida, Gainesville, Florida, USA

**Keywords:** *Arabidopsis*, branched-chain amino acid (BCAA), dihydroxyacid dehydratase (DHAD), gametogenesis, root development, salt stress.

## Abstract

Dihydroxyacid dehydratase (DHAD) catalyses a key step in the branched-chain amino acid (BCAA) biosynthetic pathway that exists in numerous organisms, including bacteria, fungi, and plants, but not humans. In *Arabidopsis thaliana*, DHAD is encoded by a single gene (*AT3G23940*), but its biological function in controlling plant development remains uncharacterized. In this study, we showed that *DHAD* is highly expressed in most vegetative and reproductive tissues. It is an essential gene, and complete disruption caused partial sterility in both male and female gametophyte phases. In addition, reduced expression of *DHAD* in knockdown mutants resulted in a reduction in the accumulation of all three BCAAs in roots and, as a consequence, led to a shorter root phenotype, which could be restored by an exogenous supplement of free BCAAs. Interestingly, the knockdown mutants became hypersensitive to salt stress, not to heavy metal stress, implying that BCAAs may act as osmolytes in salt tolerance. This would be the second amino acid shown to confer such a function in addition to the well-documented proline. Our results provide evidence that BCAA biosynthesis plays important roles in gametophyte and root development, and BCAA homeostasis contributes to the adaptation of *Arabidopsis* to salinity stress.

## Highlight

Molecular characterization of dihydroxyacid dehydratase in *Arabidopsis* reveals its important roles in gametophyte and root development, as well as involvement in salinity stress resistance.

## Introduction

Isoleucine (Ile), valine (Val), and leucine (Leu) are grouped as branched-chain amino acids (BCAAs), as they all contain short branched hydrocarbon residues. Research interest in the biosynthetic pathway of BCAAs in plants was motivated by two main reasons. First, like animals, human are unable to synthesize BCAAs *de novo*, and have to obtain them through diet or other sources (Jander and Joshi, 2009, 2010). Second, some enzymes involved in BCAA biosynthesis are targets of herbicide applications (Tan *et al.*, 2006).

The enzymes participating in BCAA biosynthesis are widely conserved in bacteria, fungi, and plants. Threonine deaminase (TD) catalyses the first and committed step towards Ile biosynthesis by converting threonine into 2-oxobutanoate (Mourad and King, 1995; Joshi *et al.*, 2006). When condensed with one molecule of pyruvate and following four more enzymatic reactions, 2-oxobutanoate is transformed into Ile (Yu *et al.*, 2013). A unique feature of BCAA biosynthesis is that a single set of four enzymes catalysing Ile production are also utilized in Val biosynthesis. These enzymes are acetohydroxyacid synthase (AHAS; EC 4.1.3.18), ketolacid reductoisomerase (KARI; EC 1.1.1.86), dihydroxyacid dehydratase (DHAD: EC 4.2.1.9) and branched-chain aminotransferase (BCAT; EC 2.6.1.42). The last intermediate of Val biosynthesis, 3-methy-2-oxobutanoate, also serves as the initial substrate in the pathway toward Leu biosynthesis. After undergoing four enzymatic steps, including condensation, isomerization, decarboxylation and transamination, the intermediate is ultimately converted to Leu (de Kraker *et al.*, 2007; Knill *et al.*, 2008; He *et al.*, 2009; He *et al.*, 2010; He *et al.*, 2011). To date, virtually all enzymatic activities required for BCAA biosynthesis have been detected in plants, including the model plant *Arabidopsis*. However, not all genes encoding these enzymes have been characterized in detail (Binder, 2010). For example, very little is known about KARI and DHAD, although the corresponding genes in *Arabidopsis* have been annotated for years based on sequence similarity to microbial homologues (Binder, 2010).

Proline accumulation has long been recognized to play an important role in osmotic regulation under a wide range of abiotic stresses (Liu and Zhu, 1997; Verbruggen and Hermans, 2008; Mattioli *et al.*, 2009; Hayat *et al.*, 2012). Partial deficiency of proline in mutants impaired in proline biosynthesis, such as *p5cs1*, caused plants to become hypersensitive to salt stress (Székely *et al.*, 2008). However, it has been recognized that proline was not the only overproducing amino acid under osmotic stresses in some studies. In fact, the levels of other amino acids, particular three types of BCAAs, could be elevated to levels comparable to proline or even greater (Jander and Joshi, 2009; Joshi *et al.*, 2010). This suggests that BCAA biosynthesis might also be involved in plant stress tolerance, but no direct evidence has been obtained to support this hypothesis.

To investigate the important roles of *DHAD* in regulating plant development and stress tolerance, we characterized the physiological alterations in *Arabidopsis DHAD* knockout and knockdown mutants. The lethality of the knockout mutants was identified and is partially caused by impairment in male and female gamete development. In the knockdown mutants, the levels of all three types of BCAA were reduced in roots, leading to a shorter root phenotype. More interestingly, the knockdown mutants exhibited higher sensitivity to salt stress, providing evidence for the first time that BCAA homeostasis plays a role in plant salt tolerance.

## Materials and methods

### Plant materials

Seeds of *Arabidopsis thaliana* ecotype Columbia (Col-0, CS3879) and SALK mutant lines *dhad-1* (SALK_062347), *dhad-2* (SALK_075098/SALK_130404), *dhad-3* (WiscDsLoxHs135_03D), and *dhad-4* (WiscDsLoxHs184_11A) were obtained from the *Arabidopsis* Biological Resource Center (ABRC). Seeds of the *lib* mutant were kindly offered by Dong Liu (Yu *et al.*, 2013). The seeds were surface sterilized using 70% ethanol for 1min, 50% bleach for 10min, and washing four times with sterilized water for 1min each time. Seeds were germinated on half-strength Murashige & Skoog (half MS) agar medium containing 1% sucrose, and cultivated in a growth chamber with a photoperiod of 16h light at 22°C and 8h dark at 20°C, and 70% humidity, for 7 days. The seedlings were transferred to soil and grown under the same conditions. For salt and nickel stress experiments, *Arabidopsis* seedlings were germinated and grown on the same half MS medium containing different concentrations of Na^+^ and Ni^2+^ by adding NaCl and NiSO_4_, respectively. The root length was measured with a ruler and the number of lateral roots was recorded using dissecting light microscopy.

### DNA extraction and genotyping

A rapid genomic DNA extraction protocol was utilized in the study. Briefly, pieces of leaf tissue collected from 3-week-old plants were grounded in extraction buffer (200mM Tris/HCl pH 8.0, 250mM NaCl, 25mM EDTA, and 0.5% SDS) using a small blue pestle. After centrifugation at 14,000rpm for 5min at 4°C, the supernatant was transferred into a new tube and precipitated by adding an equal amount of isopropanol. After centrifugation at 14,000rpm for 5min at 4°C, DNA pellets were rinsed with 70% ethanol, air dried for 10min, and finally resuspended in 1XTE buffer (10mM Tris/HCl pH 8.0 and 0.1mM EDTA).

PCR reactions were performed in a total volume of 10 μl with 5 μl 2X Green GoTag Master mix (Promega, http://www.promega.com/), 1 μl DNA template, 0.2 μl 10ng μl^–1^ forward and reverse primers, and 3.6 μl ddH_2_O. The PCR programme was as follows: 30 s at 94°C, 30 s at 50–55°C (annealing temperature dependent on the specific primer pairs; Supplementary Table S1), and 1min kb^–1^ at 72°C for a total of 37 cycles. T-DNA-specific primers for SALK and WiscDsLoxHs lines were LBa1 and LB4, respectively. *dhad-1* and *dhad-2* were genotyped using LBa1/DHAD-1RP and DHAD-1LP/DHAD-1RP, *dhad-3* was genotyped using LB4/DHAD-2RP and DHAD-2LP/DHAD-2RP, and *dhad-4* was genotyped using LB4/DHAD-3RP and DHAD-3LP/DHAD-3RP.

### RNA extraction and quantitative real-time PCR

RNA was extracted from leaves and roots using an RNeasy plant mini kit (Qiagen, http://www.qiagen.com/). cDNA was synthesized from 5 μg of total RNA using the ProtoScript® First Strand cDNA Synthesis Kit (New England Biolabs) following the manufacturer’s instructions. Quantitative real-time PCR was conducted on the ABI 7500 real-time PCR system (Applied Biosystems) according to the manufacturer’s instructions. PCR reactions were performed in a total volume of 20 μl containing 10 μl iTaq Universal SYBR Green Supermix (Bio-Rad), 0.5 μl 10ng μl^–1^ forward and reverse primers (DHAD-F and DHAD-R), and 2 μl of diluted cDNA. The PCR programme was as follows: 95°C for 30 s, followed by 40 cycles of 95°C for 5 s and 60°C for 34s. The levels of gene expression were calculated using the comparative CT method and the expression of *ACTIN2* was used as an internal control for data normalization. Data shown were the averages of three independent experiments and the primer sequences are listed in Supplementary Table 1.

### Promoter-GUS fusion, *Arabidopsis* transformation, and GUS assay

The *Arabidopsis DHAD* promoter (from −965 to −1bp) was amplified from genomic DNA using primers DHAD-1L and DHAD-1R, and the PCR product was cloned into pGEM-T vector (Promega). Once accuracy was verified by sequencing, the promoter sequence was subcloned into pCAMBIA1305 using SacI and NcoI restriction sites to produce the *Promoter*_*DHAD*_ *::GUS* construct (GUS, β-glucuronidase).

The construct was transformed into *Agrobacterium tumefaciens* strain GV3101 and subsequently transformed into plants through the floral dipping method (Clough and Bent, 1998). Transgenic plants were selected using 50ng µl^–1^ hygromycin.

Histochemical GUS assays were performed on homozygous T3 transgenic plants as described previously (Jefferson *et al.*, 1987).

### Amino acid analysis

Free amino acids were extracted using a methanol and chloroform method from leaves (100mg) and roots (50mg) (Colebatch *et al.*, 2004), and profiled using an HPLC-based pre-column derivatization protocol (Schuster *et al.*, 2006).

## Results

### AtDHAD is encoded by a single gene that is constitutively expressed

**Fig. 1.**
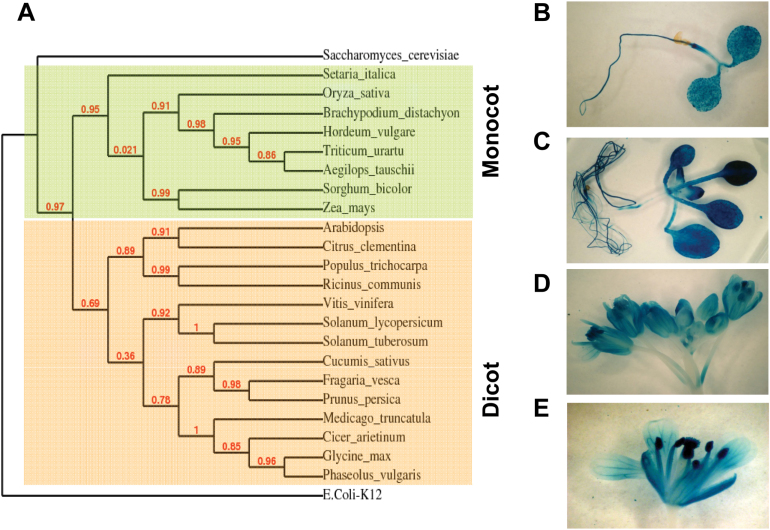
Phylogenetic analysis and tissue-specific expression of *Arabidopsis DHAD*. (A) A phylogenetic tree was constructed by the neighbour-joining method using 1000 bootstraps. The DHAD phylogeny was rooted with the *Escherichia coli* DHAD orthologue. (B–E) Histochemical assay of GUS activity using *AtDHAD* promoter-GUS plants: (B) 3-day-old seedling; (C) 10-day-old seedling; (D) and (E) flowers of 3-week-old plants. This figure is available in colour at *JXB* online.

Based on high sequence similarity to orthologues in other organisms, the gene encoding DHAD in *Arabidopsis* has been assigned to AT3G23940 in the Plant Metabolic Pathway Database (www.PlantCyc.org) and in other reviews (Jander and Joshi, 2009; Binder, 2010). To evaluate the evolutionary relationships of *DHAD* across different plant organisms, a phylogenetic tree based on amino acid sequences was constructed. The result showed that *DHAD* genes from monocots and dicots formed two distinct clades, indicating that although the sequences were fundamentally conserved, *DHAD* genes underwent subsequent divergence accompanied by the evolution of these groupss (Fig. 1A). It is also worth noting that *DHAD* seems to occur as a single-copy gene in all plant species investigated, implying that there is selection pressure to preserve *DHAD* genes as singletons following genome-wide or small-scale duplication events during evolution.

To investigate the tissue-specific expression of *DHAD*, histochemical assay of GUS activity was performed using transgenic *Arabidopsis* containing a *DHAD* promoter-GUS construct. Of 20 independent transgenic lines investigated, 19 displayed similar patterns of GUS expression. Strong GUS staining was already observed in cotyledons and roots at 3 days after germination (Fig. 1B). At 10 days, intense staining was found in developing leaves and roots (Fig. 1C). In the flower, GUS staining was observed in nearly all the organ parts, including sepals, petals, anthers, and pistils (Fig. 1D, E).

### Identification of T-DNA mutants of *AtDHAD*

**Fig. 2.**
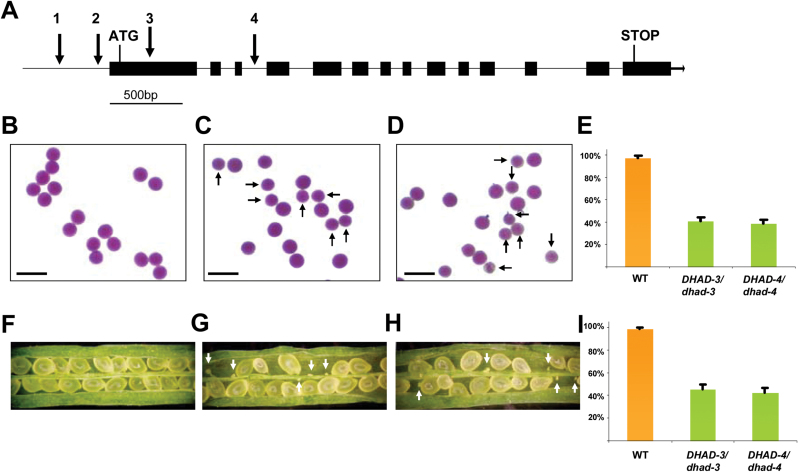
*AtDHAD* gene structure, mutation sites, and phenotype of loss-of-function mutant alleles. (A) Exon-intron structure and the T-DNA insertion sites. Each arrow indicates a T-DNA insertion site in different mutant alleles: 1, *dhad-1*; 2, *dhad-2*; 3, *dhad-3*; 4, *dhad-4*. (B–D) Alexander staining of pollen grains from the WT (B), *DHAD-3/dhad-3* (C), and *DHAD-4/dhad-4* plants (D). Arrows highlight the smaller pollen grains. Bar, 100 μm. (E) Statistical counting of the aborted pollen grains. Error bars indicate standard error. (F–H) Examination of open siliques from WT (F), *DHAD-3/dhad-3* (G), and *DHAD-4/dhad-4* plants (H). Arrows highlight the aborted ovules. (I) Statistical counting of the aborted ovules. Error bars indicate standard error. This figure is available in colour at *JXB* online.

The *AtDHAD* gene contains 14 exons and 13 introns, and encodes a putative 608 amino acid protein (Fig. 2A). To investigate the biological importance of *AtDHAD* in plant development and stress tolerance, we obtained five T-DNA insertion lines from the SALK and WiscDsLox collections (Alonso *et al.*, 2003; Woody *et al.*, 2007), named hereinafter *dhad-1* (SALK_062347), *dhad-2* (SALK_075098/SALK_130404), *dhad-3* (WiscDsLoxHs135_03D), and *dhad-4* (WiscDsLoxHs184_11A). The insertions sites of all these mutant lines were verified by sequencing the junctions of the gene/T-DNA. It should be noted that the sequencing results showed that SALK_075098 and SALK_130404 harbour T-DNA at the same location, so only SALK_130404 was chosen for further study. The insertions of *dhad-1* and *dhad-2* reside in the putative promoter or 5’UTR regions with 273bp and 88bp upstream of the start codon, respectively. The insertions of *dhad-3* and *dhad-4* locate in exon 1 and intron 3, respectively (Fig. 2A).

### *AtDHAD* is an essential gene in *Arabidopsis*

The heterozygous plants of *dhad-3* and *dhad-4* did not show any apparent phenotypes under normal growth conditions, however we failed to find any homozygous plants from a large population of T2 progeny (Table 1), suggesting that loss of function of *DHAD* caused lethality. Meanwhile, segregation distortions were observed in both *dhad-3* and *dhad-4* alleles, with the ratios of wild-type (WT) to heterozygous plants far more different from the expected 1:2 (indicative of embryo lethality) or 1:1 (indicative of single gamete lethality), suggesting that abortion might occur in the development of both male and female gametophytes (Table 1).

**Table 1.** Genetic segregation and transmission efficiency of *dhad-3* and *dhad-4* alleles in reciprocal crosses^a^

Parental genotypes		Progeny genotypes			TE (%)^b^
Female	Male	WT	HT	HH	
*DHAD-3/dhad-3*	*DHAD-3/dhad-3*	234	165	0	–
WT	*DHAD-3/dhad-3*	312	146	–	46.8^c^
*DHAD-3/dhad-3*	WT	246	108	–	43.9^d^
*DHAD-4/dhad-4*	*DHAD-4/dhad-4*	232	158	0	–
WT	*DHAD-4/dhad-4*	273	121	–	44.3^c^
*DHAD-4/dhad-4*	WT	208	86	–	41.3^d^

^a^ TE, transmission efficiency; HT, heterozgyous; HH, homozygous.

^b^ TE (%) = (observed number of HT alleles/observed number of WT alleles) × 100 (Ebel *et al.*, 2004).

^c^ Male TE.

^d^ Female TE.

To ascertain the origin of lethality from male, female, or both gametes, the reciprocal cross-pollination study was performed and the transmission efficiency (TE) through both male and female was calculated. As shown in Table 1, when heterozygous *dhad-3* or *dhad-4* plants were used as pollen donors, the TEs of *dhad* were 46.8% and 44.3% for *dhad-3* or *dhad-4*, respectively, indicating that a proportion of *dhad* male gametes was aborted. Likewise, the female TEs were 43.9% and 41.3% for *dhad-3* or *dhad-4*, respectively, indicating that the female gametophyte was affected to a similar extent to the male gamete (Table 1).

To clarify the effects of disruption of *DHAD* on male gamete development, Alexander staining was used to observe pollen viability and morphology (Alexander, 1969). For the WT, all pollens grains were uniformly shaped and sized (Fig. 2B). In comparison, although largely red stained, ~40% of pollen grains from *DHAD-3/dhad-3* (40.6%; *n* = 1225) and *DHAD-4/dhad-4* (38.3%; *n* = 1335) were smaller in size (Fig. 2C–E). Given that the percentage of pollen grains displaying morphological alterations correlated well with the observed frequency of male gametophytes with the mutant genotype (Table 1), we inferred that the genotype of the small pollen grains was *dhad*, and that such abnormality may cause pollen grains that are less- or non-viable compared to WT pollen (Fig. 2C, D, E).

According to the TE data, female gametophyte development was also impaired in the *dhad* mutants (Table 1). This was further supported by examination showing that small, white, unfertilized ovules were substantially observed in the developing siliques of *DHAD-3/dhad-3* and *DHAD-4/dhad-4* (Fig. 2F–H). The frequencies of aborted ovules were 45.6% (*n* = 542) and 42.1% (*n* = 503) for *DHAD-3/dhad-3* and *DHAD-4/dhad-4*, respectively (Fig. 2I). This is consistent with the values calculated from TE analysis (Table 1). In addition, it should be noted that although both male and female gamete development resulted in mostly sterile gametes, a small proportion were still viable; this is opposed to the genetic analysis in which none of the homozygous mutant plants could be recovered from F_2_ progeny, implying that loss of function of *DHAD* may also cause embryo lethality.

### Decreased expression of *AtDHAD* led to retarded root growth

In contrast to the lethality of *dhad-3* and *dhadh-4* knockouts, homozygous plants were successfully obtained from self-pollinating progenies of heterozygous *dhad-1* and *dhad-2*, the alleles containing the insertions in the promoter or 5’UTR regions of *DHAD* (Fig. 1A). Real-time quantitative PCR analysis showed that the expression levels of *DHAD* in homozygous *dhad-1* and *dhad-*2 were decreased to 36% and 25% compared to WT sibling plants (Supplementary Figure S1A), indicating that T-DNA insertions at the promoter or 5’UTR regions downregulated the expression of *DHAD*.

**Fig. 3.**
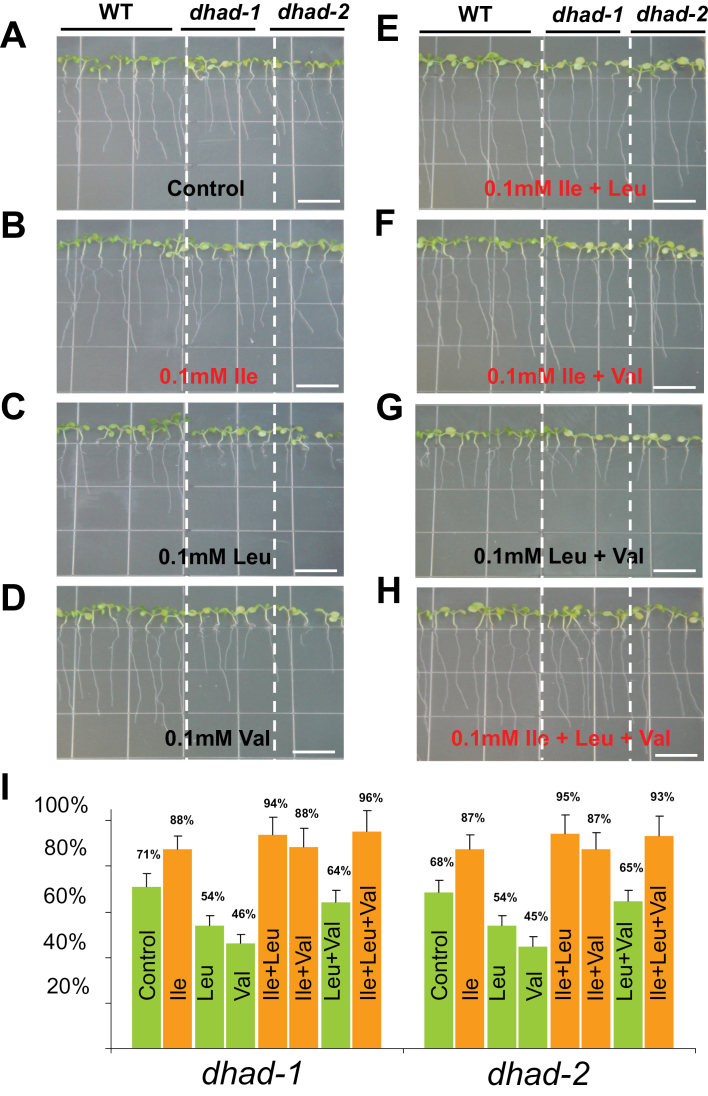
Rescue of the shorter root phenotype in *dhad-1* and *dhad-2* mutants by exogenous application of Ile. (A–H) 10-day-old WT, *dhad-1*, and *dhad-2* seedlings grown on half MS medium supplemented with 0.1mM Ile (B), 0.1mM Leu (C), 0.1mM Val (D), 0.1mM Ile/Leu (E), 0.1mM Ile/Val (F), 0.1mM Leu/Val (G), and 0.1mM Ile/Leu/Val (H). Bar, 10mm. (I) Root length of the mutants was measured and shown as a percentage relative to that of the WT grown in the same conditions. Error bars indicate standard error. This figure is available in colour at *JXB* online.

When grown in soil, homozygous plants did not show any obvious phenotypes, such as morphological appearance, growth rate, and bolting time compared to WT (Supplementary Figure S1B). However, when seeds were germinated on half MS medium and grown vertically for 10 days, the average root length in both *dhad-1* and *dhad-2* was significantly shorter than WT (Fig. 3A), and the lateral root density was also decreased (Supplementary Figure S2), suggesting that both primary and lateral root development were affected. To clarify whether the shorter root phenotype was caused by the deficiency of free BCAAs, plants were grown in medium exogenously supplied with each individual type or a variety of combinations of two or all three BCAAs. Interestingly, it was observed that when supplied with Ile (Fig. 3B), not Leu (Fig. 3C) or Val (Fig. 3D), the root length in mutants was restored to a level similar to WT, suggesting that the decreased root growth might be caused by the reduced level of Ile in mutants. It is further shown that any combinations containing Ile could rescue the root-growth phenotype (Fig. 3E, F, and H). It is worth mentioning that exogenous application of Leu caused cellular toxicity (Fig. 3C), and this toxicity was further exaggerated when Leu and Val were added together (Fig. 3G). All the above-described root-growth assays were independently repeated three times, and root growth rates are summarized and quantified in Fig. 3I.

A range of concentrations of 2-oxobutanote were exogenously applied in the growth medium. This was to test the possibility that the potential buildup of 2-oxobutanote, the Ile biosynthetic intermediate, will harm root growth in *dhad* knockdown mutants. As shown in Supplementary Figure S3, up to 0.5mM 2-oxobutanoate did not yield any distinct responses in *dhad* mutants compared to the WT, indicating that either the stunted root growth was not related to the cellular concentration of 2-oxobutanoate, or alternatively that the toxicity of 2-oxobutanoate on plant growth is extremely minimal.

To corroborate that the shorter root phenotype correlated with the reduced level of BCAAs, free amino acids were quantified in root and leaf tissues. It is noted that since both alleles, *dhad-1* and *dhad-2*, exhibited identical phenotypes in root-length assays, only *dhad-2* was used in amino acid analysis. As shown in Table 2, in roots, the abundances of all three BCAAs in *dhad-2* showed a statistically significant reduction compared to the WT, suggesting that the decreased gene dosage of *DHAD* could limit the rate of metabolic flux to form the end products, BCAAs. In addition, the contents of two precursor amino acids, aspartate and threonine, were moderately increased, suggesting that the partial blockage of BCAA biosynthesis could lead to the ectopic buildup of the precursor intermediates. By comparison, in leaves, no amino acids showed significant changes (Table 2), consistent with the indistinguishable phenotypes of the mutants and WT (Supplementary Figure S1B). Taken together, these results indicate that the decreased expression of *DHAD* resulted in partial deficiency in BCAA biosynthesis, and subsequent impairment of root development.

**Table 2.** Amino acid contents of roots and leaves in the WT and *dhad-2* ^a,b,c^

Amino acid	Roots		Leaves	
	WT	*dhad-2*	WT	*dhad-2*
Alanine	13.76±0.49	12.43±0.15	14.85±0.32	15.31±1.32
Arginine	0.54±0.02	0.45±0.03	2.11±0.54	2.61±0.18
Asparagine	24.31±2.25	33.74±1.19 ^a^	31.16±0.68	27.27±1.51
Aspartate	9.38±0.15	11.96±0.26 ^a^	24.77±0.73	26.32±0.52
Glutamine	38.32±0.05	44.99±1.26 ^a^	96.08±0.14	91.23±1.71
Glutamate	230.65±0.42	272.93±11.68	631.25±38.44	562.25±14.34
Glycine	3.65±0.15	3.14±0.02	20.61±4.48	16.3±0.71
Histidine	1.33±0.03	1.42±0.06	1.42±0.12	1.11±0.11
Isoleucine	0.93±0.01	0.66±0.03 ^b^	0.49±0.01	0.46±0.01
Leucine	1.35±0.04	0.96±0.04 ^b^	0.66±0.01	0.55±0.05
Lysine	1.1±0.01	0.91±0.01 ^b^	0.53±0.03	0.61±0.10
Methionine	3.19±0.07	2.78±0.07	1.01±0.07	0.79±0.02
Phenylalanine	0.51±0.03	0.47±0.01	0.64±0.03	0.73±0.02
Proline	16.22±2.00	18.55±0.5	21.03±0.79	25.08±3.00
Serine	15.74±1.38	19.2±0.4	37.95±0.57	42.81±2.20
Threonine	6.71±0.17	9.8±0.32 ^a^	5.48±0.03	6.54±0.59
Tyrosine	0.46±0.02	0.39±0.03	0.31±0.02	0.31±0.01
Valine	2.64±0.01	2.21±0.05 ^b^	2.27±0.04	2.13±0.15

^a^ Values significantly higher that the WT (*P* < 0.05), Student’s *t*-test.

^b^ Values significantly lower that the WT (*P* < 0.05), Student’s *t*-test.

^c^ Amino acid contents are given in pmol mg FW^–1^ (FW, fresh weight). Data are the mean ± SD of three replicates.

### Decreased expression of *DHAD* resulted in increased sensitivity to salt stress

**Fig. 4.**
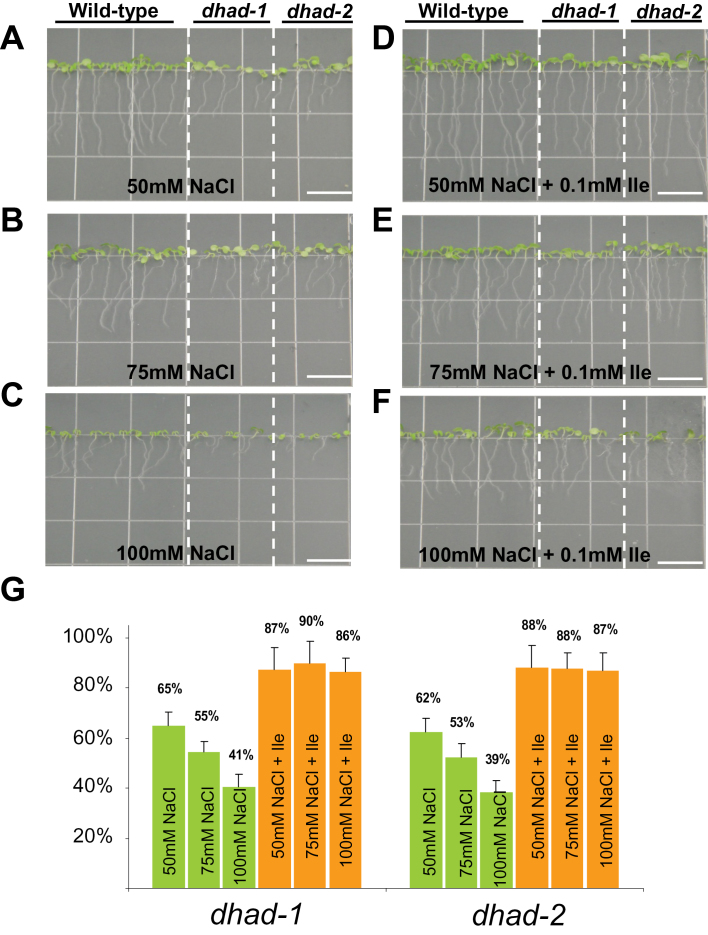
*dhad-1* and *dhad-2* are hypersensitive to salt stress. (A–F) 10-day-old WT, *dhad-1*, and *dhad-2* seedlings grown on half MS medium supplemented with different levels of NaCl (A–C) and 0.1mM Ile (A–F). Bar, 10mm. (G) Root length in the mutants was measured and shown as a percentage relative to that in the WT grown in the same conditions. Error bars indicate standard error. This figure is available in colour at *JXB* online.

Previous studies have shown that the accumulation of BCAAs was greatly elevated under abiotic stresses (Joshi and Jander, 2009; Joshi *et al.*, 2010), suggesting that BCAA homeostasis may contribute to the multifaceted stress tolerance systems in plants. To test the possibility that reduction in BCAA abundance may lead to hypersensitivity to abiotic stress, the effects of salt stress on root growth were investigated. As shown in Fig. 4A, with a low concentration of Na^+^ (50mM), the root length of *dhad* knockdown mutants was 62–64% of that in the WT (Fig. 4G), which is lower than the 68–71% without salt treatment (Fig. 3A and 3I). When Na^+^ concentration increased to 75mM, the ratio of the root length between the mutant and WT became 53–55% (Fig. 4B and 4G), and further decreased to 39–41% after treatment with 100mM Na^+^. This indicates that *dhad* knockdown mutants exhibited greater sensitivity compared to the WT towards increasing concentration of Na^+^. More importantly, the sensitivity to Na^+^ treatment under different concentrations could be restored to similar levels as in the WT when 0.1mM Ile was supplied exogenously. These results suggest that the high sensitivity of *dhad* to Na^+^ stress can be attributed to the reduced level of Ile (Fig. 4D–G).

**Fig. 5.**
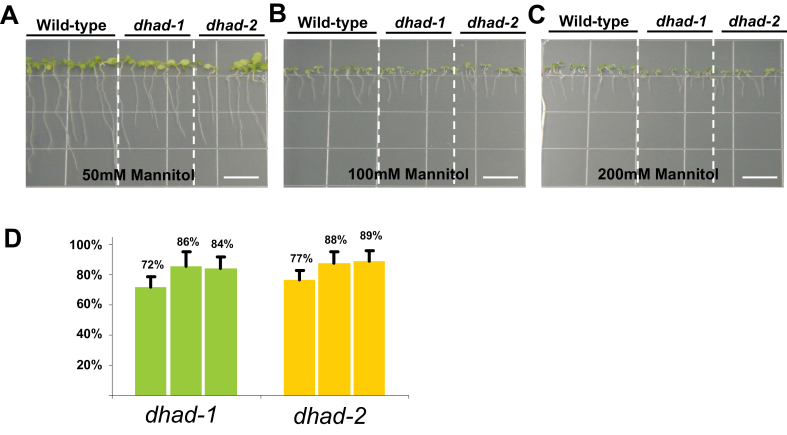
*dhad-1* and *dhad-2* do not show hypersensitivity to mannitol stress. (A–C) 10-day-old WT, *dhad-1*, and *dhad-2* seedlings grown on half MS medium supplemented with different levels of mannitol. Bar, 10mm. (D) Root length in mutants was measured and is shown as a percentage relative to that in the WT grown in the same conditions. Error bars indicate standard error. This figure is available in colour at *JXB* online.

The hypersensitive response to salinity stress may be directly derived from salt ionic toxicity, or alternatively represent a universal reaction to osmotic stress. To ascertain these two possibilities, *dhad* knockdown mutants were treated with increased concentrations of mannitol. As shown in Fig. 5, with a low concentration of mannitol, the root length of *dhad* mutants was 72–77% of that in the WT, which is comparable to the 68–71% without any treatment (Fig. 3A and 3I). However, with increased concentrations of mannitol, the ratio of root length between the mutant and WT was increased to 84–89%, which is clearly different from the salt response, indicating that there is not an exaggerated effect under osmotic stress. Therefore, we conclude that hypersensitivity in *dhad* to salinity stress can be attributed to the salt ionic toxicity.

**Fig. 6.**
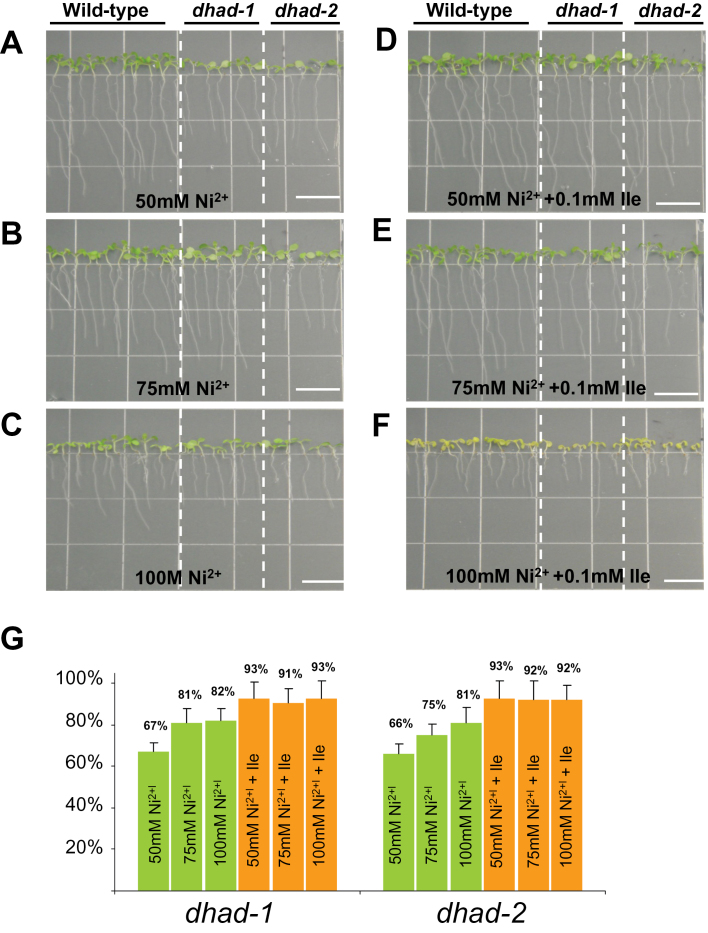
*dhad-1* and *dhad-2* do not show hypersensitivity to Ni^2+^ stress. (A–F) 10-day-old WT, dhad-1, and dhad-2 seedlings grown on half MS medium supplemented with different levels of NiSO4 (A–C) and 0.1mM Ile (A–F). Bar, 10mm. (G) Root length in mutants was measured and is shown as a percentage relative to that in the WT grown in the same conditions. Error bars indicate standard error. This figure is available in colour at *JXB* online.

The inhibition of root growth of *dhad*, by salt stress, might also reflect a common response to various abiotic stresses. To test this possibility, the root-growth assays were conducted under different concentrations of Ni^2+^ treatment, which represents a type of heavy metal stress. As shown in Fig. 6, the mutants did not exhibit the signs of high sensitivity to the increased concentrations of Ni^2+^. In contrast, the ratios of root length between mutants and WT became slightly increased under elevated concentrations of Ni^2+^. This suggests that the high sensitivity to salt stress in *dhad* is quite specific.

### The *lib* mutant shows the same pattern toward increased sensitivity to salt stress

**Fig. 7.**
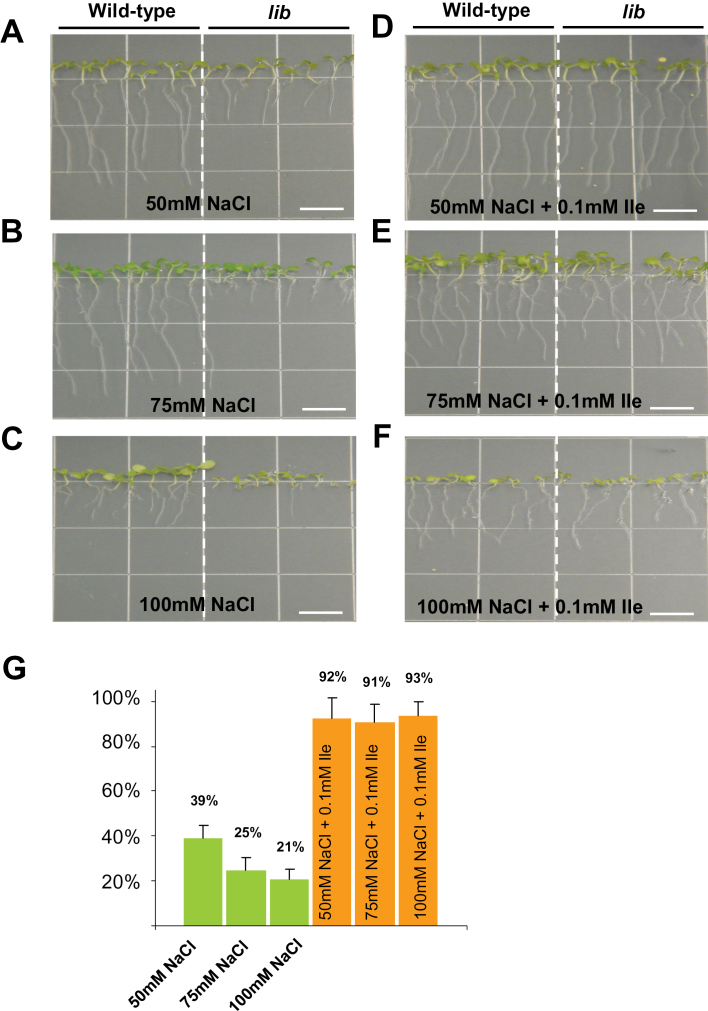
The *low isoleucine biosynthesis* (*lib*) mutant is hypersensitive to salt stress. (A–F) 10-day-old WT or *lib* seedlings grown on half MS medium supplemented with different levels of NaCl (A–C) and 0.1mM Ile (A–F). Bar, 10mm. (G) Root length in the mutants was measured and shown as a percentage relative to that in the WT grown in the same conditions. Error bars indicate standard error. This figure is available in colour at *JXB* online.

To further establish the functional role of Ile biosynthesis in tolerance to salinity stress, the root-growth assays were conducted with *lib* (Yu *et al.*, 2013). As shown in Fig. 7, similar to *dhad* alleles, *lib* shows higher sensitivity toward increased salt concentration, and this hypersensitivity could be rescued by exogenous application of Ile, unambiguously indicating that the Ile biosynthetic pathway plays a critical role in salt tolerance.

## Discussion

As the essential components of protein synthesis and also as important signalling molecules, BCAAs have been thought to regulate numerous aspects of plant growth required for plant survival. However, little information is available about specific roles of BCAAs in plant growth and development as well as interaction with the environment. In recent years, extensive studies in *Arabidopsis* have led to the identification of most of the genes involved in BCAA biosynthesis. To date, only two genes, *KARI* and *DHAD*, remain functionally uncharacterized. In this study, we identified that null mutations of *DHAD* caused partial abortion in both male and female gametogenesis. This is reminiscent of the loss of function of *LeuD3* and *IPMDH2/IPMDH3*, involved in Leu biosynthesis, where the lethality was detected in female or male gamete development, respectively (He *et al.*, 2009; He *et al.*, 2010). The underlying causes of lethality in distinct organs of different mutants include (i) different organs having exceptional requirements for the *de novo* biosynthesis of certain types of BCAAs; (ii) different biosynthetic intermediates having dissimilar abilities or requirements to be transported from sporophytic into reproductive tissues; and (iii) the potential cellular toxicities resulting from the abnormal accumulation of biosynthetic intermediates having different effects on plant development.

The decreased expression of *DHAD* resulted in a simultaneous reduction in the levels of the three BCAAs in roots, but not in leaves (Table 2), indicating that *DHAD* may exert rate-limiting control in regulating BCAA biosynthesis in a tissue-specific manner. However, the possibility of the existence of an alternative pathway for BCAA biosynthesis in leaves cannot be completely ruled out in this study. In addition, the retarded root growth could only be rescued by the exogenous application of Ile, not Leu or Val, suggesting that the impaired root growth was most probably caused by the deficiency in Ile accumulation. This result is consistent with results from a recent study in which a mutation in threonine deaminase/dehydratase catalysing the first and also committed step of Ile biosynthesis resulted in partial Ile deficiency, and consequently retarded root development (Yu *et al.*, 2013). Therefore, it is clear that Ile homeostasis plays an important role in regulating root development in *Arabidopsis*. Moreover, the perturbation of *DHAD* may lead to a buildup of metabolic intermediates. For instance, it has previously been shown that the inhibition of *AHAS* can cause toxic accumulation of ketobutyrate in *Salmonella* (LaRossa, 1987). However, in this study, no hint of the cellular toxicity caused by 2-oxobutanoate was suggested, since the exogenous application of substantial amounts of 2-ketoburate didn’t produce any distinct response in *dhad* relative to the WT. Furthermore, we observed that the concentrations of several other amino acids were affected, including increased levels of asparagine, aspartate, glutamate, and threonine, as well as a reduced level of lysine. As the precursor amino acid for Ile, the accumulation of threonine could simply be explained by potential build-up due to the blockage of downstream metabolic flux. Similarly, all the BCAAs are aspartate-derived amino acids, therefore the reduction in BCAAs could lead to a build-up of aspartate, subsequently further recruited into asparagine. However, the way in which glutamine increased and lysine decreased is unclear, suggesting that the biosynthesis of different amino acids is under the regulation of complex crosstalk, and the precise change leading to the imbalance of different amino acids deserves further investigation. In the *lib* mutant, in addition to the reduced level of Ile, only two other amino acids showed statistically significant changes in abundance; these were an increase in phenylalanine and a decrease in lysine. The consistent reduction in Ile content in both *lib* and *dhad* indicates that both genes are involved in Ile synthesis, but the distinct response of other amino acids towards the impairment in different steps along the same biosynthetic pathway suggests that the metabolic network associated with each step was slightly dissimilar. Taken together, although the possibility of the stunted root growth being a consequence of intermediate accumulation could not be completely ruled out, we speculate that the root phenotype is mostly likely caused by Ile deficiency.

Under abiotic stresses, the levels of some amino acids were significantly increased. Proline biosynthesis has been well studied and shown to play important roles in plant tolerance toward various stresses Liu and Zhu, 1997; Verbruggen and Hermans, 2008; Mattioli *et al.*, 2009; Hayat *et al.*, 2012). Disruption of proline biosynthesis renders plants hypersensitive to salinity stress (Liu and Zhu, 1997; Mattioli *et al.*, 2009). Interestingly, it has long been recognized that the accumulation of the three BCAAs exhibited more significant induction than proline in some cases of osmotic stresses, implying that BCAAs may also play a role in plant stress responses (Joshi and Jander, 2009; Joshi *et al.*, 2010). However, no functional evidence was obtained to draw such a conclusion. In this study, we found that the decreased expression of *DHAD* led to a reduction in BCAA levels and rendered plants hypersensitive to salinity stress (Table 2 and Fig. 4), providing for the first time strong evidence that BCAA homeostasis has an important impact on plant salinity tolerance. In addition, such hypersensitivity seems to be rather specific for salinity stress, since the parallel assay to test the response to osmotic stress and heavy metal stress showed an opposite result. How does the deficiency in BCAAs change plant sensitivity to salinity stress? We speculate that this is related to both aspects of fundamental functions of BCAAs, in which they serve either as substrates for stress-induced protein biosynthesis or as signalling molecules for regulating stress-responsive gene expression.

To conclude, in this work, molecular and physiological responses to altered expression of *DHAD* were characterized in *Arabidopsis*. Our results demonstrate that *DHAD* encodes an essential gene and plays important roles in both male and female gametogenesis, as well as root development. More importantly, we have observed that partial deficiency of BCAAs renders plants hypersensitive to salt stress. The result provides novel evidence concerning the long-standing hypothesis that BCAA homeostasis contributes to the adaptation of plants to salinity stress.

## Supplementary material

Supplementary data can be found at *JXB* online.

Supplementary Table S1. Primer sequences used in the study.

Supplementary Figure S1. Quantitative real-time expression and phenotypic analysis of homozygous *dhad-1* and *dhad-2*.

Supplementary Figure S2. Lateral root density of 10-day-old WT and *dhad* grown on 1/2MS medium.

Supplementary Figure S3. *dhad-1* and *dhad-2* do not show hypersensitivity to 2-oxobutanoate.

## Funding

This work was supported by funding from the National Programme on Key Basic Research Project in China (973Programme: 2014CB147301) to YH, and partly by the US National Science Foundation CAREER Award 0845162 to SC.

## Supplementary Material

Supplementary Data

## Abbreviations:

AHAS: acetohydroxyacid synthase
BCAA: branched-chain amino acid
BCAT: branched-chain aminotransferase
DHAD: dihydroxyacid dehydratase
EDTA: ethylene diamine tetraacetic acid
HPLC: high performance liquid chromatography
KARI: ketolacid reductoisomerase
Ile: isoleucine
IPMDH: isopropylmalate dehydrogenase
Leu: leucine
MS: Murashige and Skoog
PCR: polymerase chain reaction
T-DNA: transferred DNA
TD: threonine deaminase
TE: transmission efficiency
Val: valine
WT: wild-type.
