# Supplementary material for: Dihydroxyacid dehydratase is important for gametophyte development and disruption causes increased susceptibility to salinity stress in Arabidopsis
Source: J Exp Bot. 2014 Nov 13;66(3):879–88. doi: 10.1093/jxb/eru449 (PMC4321549; doi:10.1093/jxb/eru449)
Supplement: Supplementary Data [file supp_66_3_879__index.html]

Dihydroxyacid dehydratase is important for gametophyte development and disruption causes increased susceptibility to salinity stress in Arabidopsis — Dihydroxyacid dehydratase is important for gametophyte development and disruption causes increased susceptibility to salinity stress in Arabidopsis — Supplementary Data 

# Dihydroxyacid dehydratase is important for gametophyte development and disruption causes increased susceptibility to salinity stress in *Arabidopsis*

## Supplementary Data

Data files

**Files in this Data Supplement:**

- Supplementary Data - Supplementary Data
- Supplementary Data - Supplementary Data
