# Supplementary material for: Dihydroxyacid dehydratase is important for gametophyte development and disruption causes increased susceptibility to salinity stress in Arabidopsis
Source: J Exp Bot. 2014 Nov 13;66(3):879–88. doi: 10.1093/jxb/eru449 (PMC4321549; doi:10.1093/jxb/eru449)
Supplement: Supplementary Data [file supp_eru449_jexbot135830_file001.pdf]

A B

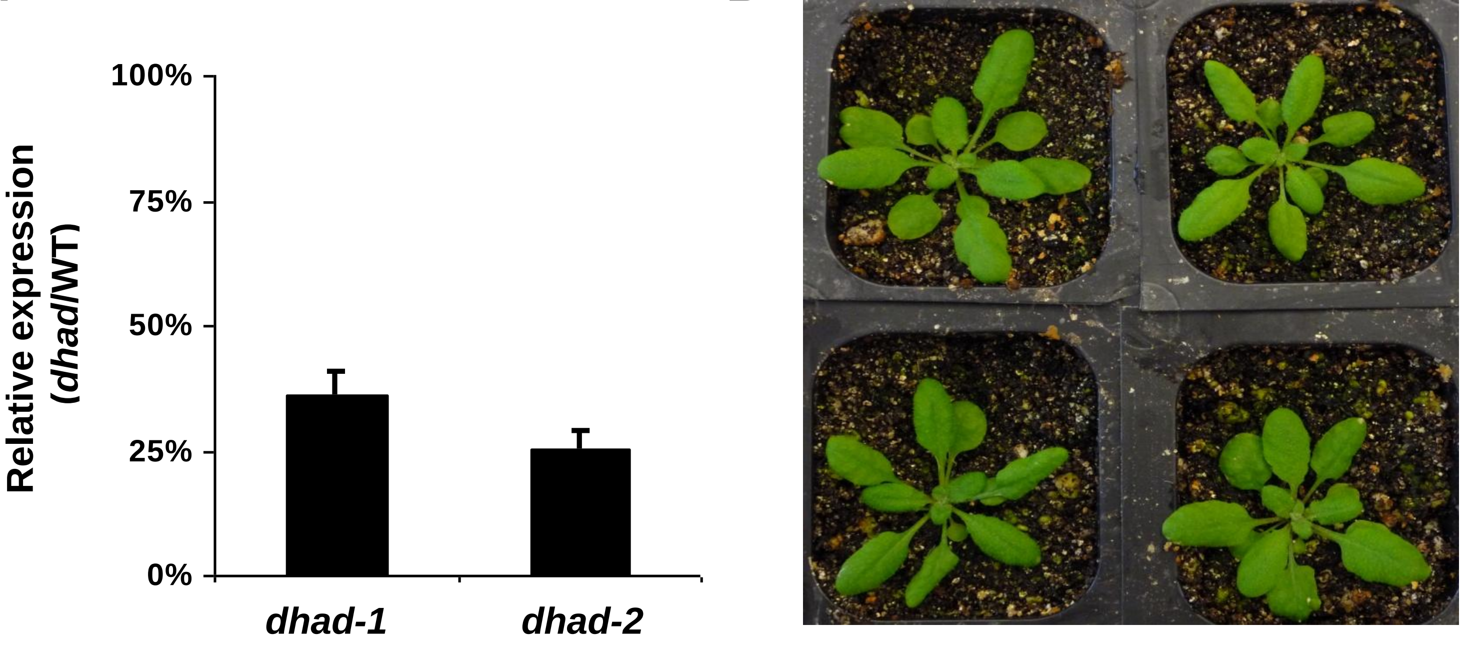

DHAD-1/DHAD-1	*dhad-1/dhad-1*
DHAD-2/DHAD-2	*dhad-2/dhad-2*

Figure S1. Quantitative real-time expression and phenotypic analysis of homozygous *dhad-1* and *dhad-2*.

A. Relative expression of *DHAD* gene in *dhad-1* and *dhad-2* homozygous mutants compared to corresponding sibling WT plants. The expression was first normalized using housekeeping gene *ACTIN* as internal standard and then the relative expression was calculated with the ratio of *dhad* versus WT. The data were shown as mean ± SD from three independently biological experiments.

B. Overall morphology of three-week-old plants for each genotype as indicated.

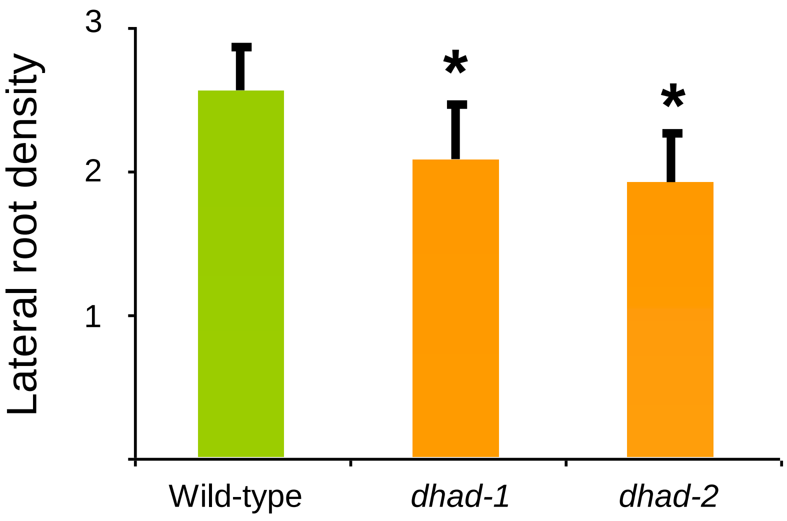

Figure S2. Lateral root density of 10-day-old WT and *dhad* grown on 1/2MS medium. The density was calculated as the number of lateral root per cm. *, statistically significant difference as determined by Student's t-test ($p<0.01$).

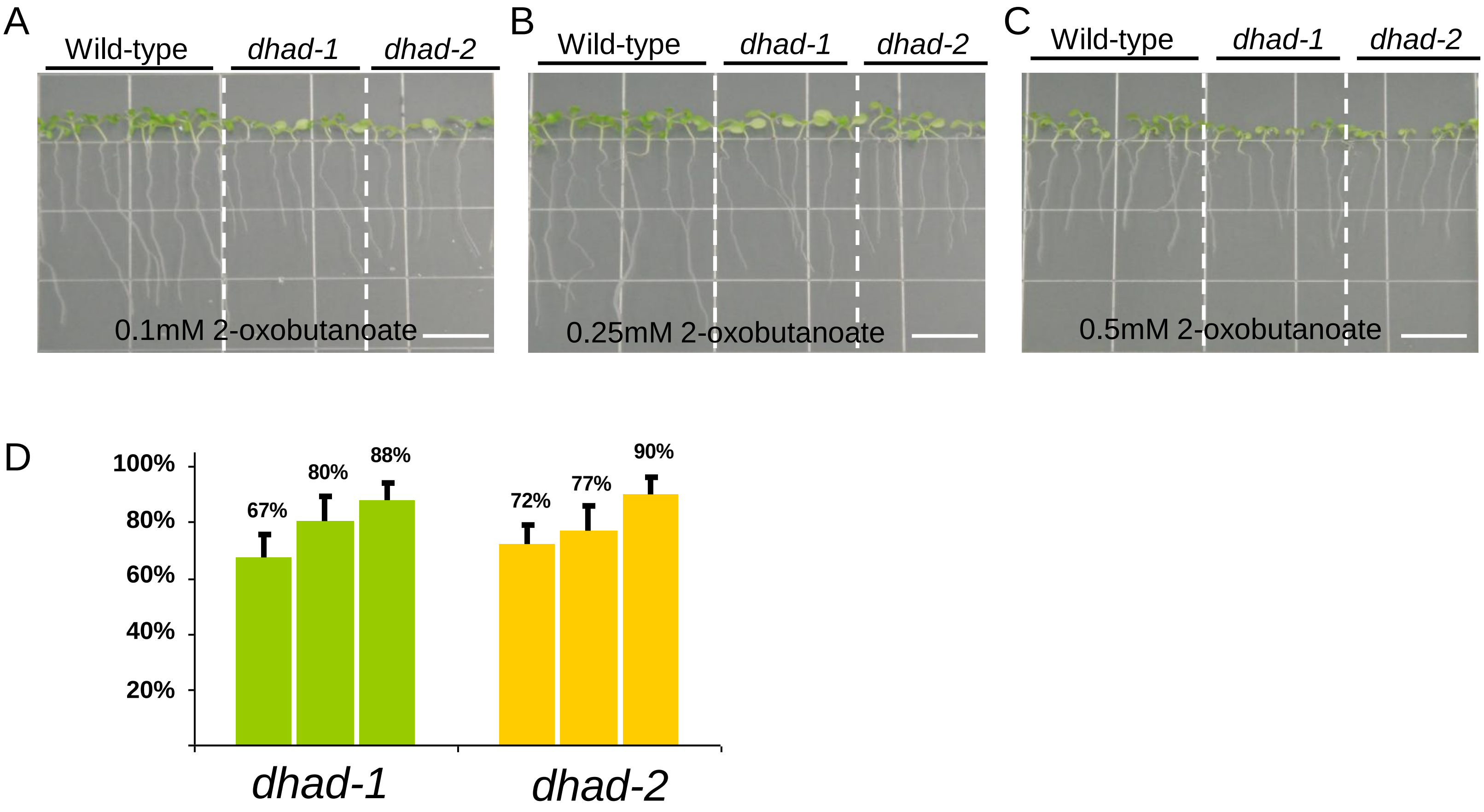

Figure S3. *dhad-1* and *dhad-2* do not show hypersensitive to 2-oxobutanoate.
(**A**)-(**C**) 10-day-old WT, *dhad-1* and *dhad-2* seedlings grown on ½ MS medium supplemented with different levels of 2-oxobutanoate. Bar: 10mm.
(**D**) Root length in mutants was measured and is shown as a percentage relative to that in WT grown on same condition. Error bars indicate standard error.
